# Mental Health Home Care program to pacients with Serious Mental Disorders

**DOI:** 10.1192/j.eurpsy.2022.1607

**Published:** 2022-09-01

**Authors:** S.M. Bañón González, N. Ogando Portilla, C. Montalvo Vico, F. Garcia Sánchez, M. Gutiérrez Rodríguez, R. Gutiérrez Labrador

**Affiliations:** 1 Hospital Infanta Sofía, Psiquiatría, Madrid, Spain; 2 Hospital Universitario de Mostoles, Psiquiatría, Madrid, Spain

**Keywords:** Mental Health Home Care, environment, Serious Mental Disorders, Multidisciplinaty

## Abstract

**Introduction:**

The Mental Health Home Care is a program whose main objective is to provide care to people with a Serious Mental Disorders with difficulties to maintain continuity of treatment and link between the different resources. It is a program that guarantees continuity of care and facilitates the link between the different rehabilitation resources. It carries out a multidisciplinary approach to the difficulties of the patient and the family.

**Objectives:**

Both analyze clinical, psychopathological and epidemiological characteristics of Serius Mental Disorders and review causes, incidence, prevalence, diagnostic, therapeutic tools and the importance of maintaining the treatment and rehabilitation in Serius Mental Disorders, because the abandonment of the treatment is a predictor of relapses.

**Methods:**

Review of the impact literature for the last five years concerning Serius Mental Disorder: prevalence, incidence, pathogenesis and its relationship with other psychiatric disorders encoded in DSM-V.

**Results:**

The program is made up of a Psychiatrist, a Clinical Psychologist, a Mental Health Nurse and two Nursing Auxiliary Care, two Social Workers and two Occupational Therapists.The responsible professional presents the patient at the program meetings. The program’s multidisciplinary team proposes an individualized treatment plan for the patient and family in the patient’s environment.

**Conclusions:**

The objective and areas of global intervention is to provide comprehensive psychiatric, psychological, social and rehabilitative support in patients with difficulty in linking to other resources, keeping the patient in a normalized community context, improving treatment compliance and making appropriate use of standardized mental health services.

**Disclosure:**

No significant relationships.

